# Incidence of venous thromboembolism in rheumatoid arthritis, results from a “real-life” cohort and an appraisal of available literature

**DOI:** 10.1097/MD.0000000000026953

**Published:** 2021-08-20

**Authors:** Alessandro Conforti, Onorina Berardicurti, Viktoriya Pavlych, Ilenia Di Cola, Paola Cipriani, Piero Ruscitti

**Affiliations:** Department of Biotechnological and Applied Clinical Sciences, University of L’Aquila, L’Aquila, Italy.

**Keywords:** arthritis, rheumatoid, thromboembolism, venous

## Abstract

Rheumatoid arthritis (RA) is associated with an increased risk of venous thromboembolism (VTE) occurrence. In this work, we assessed the incidence and predictive factors of VTE in our “real-life” cohort of RA patients. To contextualize our results, we reviewed the available literature about this topic.

We performed a retrospective analysis of prospectively followed-up patients with RA attending our Rheumatologic Clinic between January 2010 and December 2020. Each patient was investigated for VTE occurrence. Incident cases were reported as incidence proportion and incidence rate per 1000 person-years at risk. Possible predictive factors were also exploited by regression analyses. Available literature about this topic was also assessed.

In this evaluation, 347 consecutive patients without previous evidence of VTE, attending our Rheumatologic Clinic from 2010 to 2020, were studied. In our “real-life” cohort, the incidence proportion of VTE was 3.7% (2.7–4.7%) and considering over 1654 person-years, an incidence rate of 7.8 × 1000 (2.5–11.7). Exploratively assessing predictive factors in our cohort, older age (hazard ratio [HR] 1.07, 95% confidence interval [CI] 1.01–1.14, p = .015), higher body mass index (HR 1.37, 95% CI 1.04–1.80, *P* = .026), and longer disease duration (HR 1.11, 95% CI 1.03–1.20, *P* = .006) resulted to be significant predictors of VTE occurrence during the follow-up.

In our “real-life” cohort, VTE burden has been suggested in patients with RA. Comparing our results with previous data derived from randomized controlled trials and administrative data, some different findings were retrieved about incidence of VTE. Assessing predictive factors, older age, higher body mass index, and longer disease duration resulted to be significant predictors of VTE occurrence during the follow-up. Taking together these observations, a further evaluation of this issue on specific designed studies is needed to provide more generalizable results to the daily clinical practice.

## 1 Introduction

Rheumatoid arthritis (RA) is a systemic inflammatory disease associated with a significant morbidity and mortality.^[1,2]^ A recent growing body of evidence suggests the burden of the venous thromboembolism (VTE) on these patients, including both pulmonary embolism and deep venous thrombosis, as a cause of mortality and morbidity.^[2]^ In this context, it must be pointed out that VTE would be the third most frequent cardiovascular disease (CVD) with an overall annual incidence of 100 to 200 per 100,000 habitants.^[3,4]^ It may be lethal in the acute phase or lead to chronic disease and disability.^[4]^ Usually, VTE is a disease affecting the elderly; it is rare before the late adolescence.^[4]^ During RA, the pro-inflammatory *milieu* may induce a hyper-coagulation by an upregulation of procoagulant factors and a simultaneous downregulation of anticoagulant and fibrinolytic systems, thus leading to this clinical phenotype.^[5]^ The consequent disruption of the tightly regulated homeostatic control of immune and hemostatic systems may result in a prothrombotic tendency.^[7]^ However, it must be pointed out that the incidence of VTE is mainly estimated by randomized clinical trials (RCTs) and administrative data so far.^[2,5,6,7]^ In the ongoing study A3921133, the frequency of VTE and all-cause mortality was higher in patients receiving tofacitinib 10 mg twice daily than in others.^[7]^ This result furtherly bought the attention of the scientific community the possible increased risk of VTE in RA patients. However, although of relevance, the evidence, which is derived from these types of study, could not fully clarify this issue. In fact, the strict enrolment criteria of the clinical trials could not fully mirror the “real-life” scenario of patients daily admitted to rheumatologic clinics.^[8]^ Furthermore, administrative data, although very large in covering samples of individuals, may lack of some relevant clinical information, reducing their clinical significance.^[9]^ On these bases, we assessed incidence and predictive factors of VTE in our “real-life” cohort of RA patients. To contextualize our results, we reviewed the available literature about this topic.

## 2 Methods

### 2.1 Study design

In this study, we performed a retrospective analysis of prospectively followed-up, a prospective historical study, RA patients attending our Rheumatologic Clinic. The local Ethics Committee (Comitato Etico Azienda Sanitaria Locale 1 Avezzano/Sulmona/L’Aquila, L’Aquila, Italy, protocol number 000331/17) approved the study, which was performed according to the Good Clinical Practice guidelines and the Declaration of Helsinki. In reporting the results, we followed the STROBE guidelines (STROBE Checklist).

### 2.2 Settings and locations

**Figure 1:**
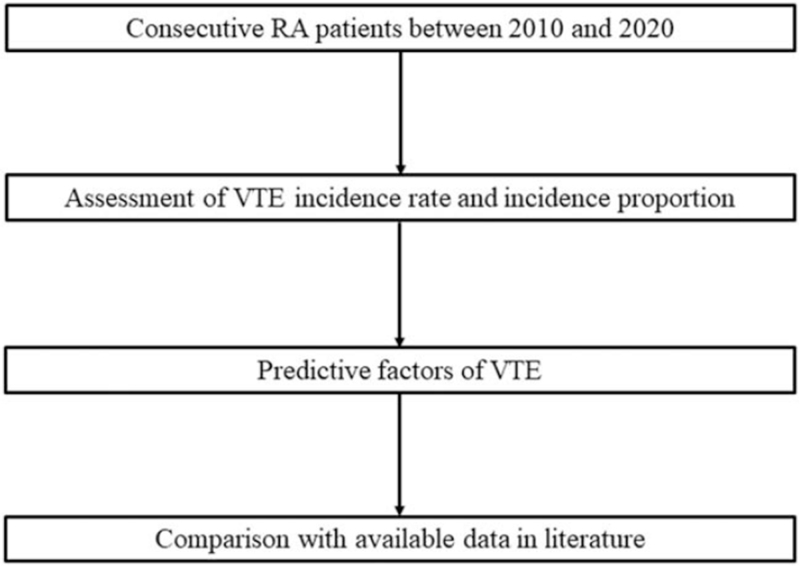
Study design.

This study was designed as retrospective analysis of patients prospectively followed-up at Rheumatologic Clinic of University of L’Aquila, L’Aquila, Italy, between January 2010 and December 2020, as reported in Fig. 1.

### 2.3 Variables to be assessed

In the present evaluation, we analyzed incidence and predictive factors of VTE in our cohort of patients with RA, as shown in Fig. 1. VTE was defined as the diagnosis of deep veins thrombosis or pulmonary embolism as per international guidelines.^[4,10]^ In all patients with VTE, a comprehensive thrombophilia screening was performed to determine the etiology of VTE, to estimate the risk of recurrence, to recommend therapy, and possible prophylactic measures. This started with a personal and family history for VTE, evaluation of acquired risk factors for VTE, and possible therapy adverse effects.^[11]^ A laboratory evaluation was performed in patients experiencing VTE assessing deficiencies of natural anticoagulant proteins (antithrombin deficiency, protein C deficiency, protein S deficiency, hyper-homocysteinemia, mutation of factor II [F2, G20210A mutation], factor V-Leiden [F5, G1691A mutation], lupus anticoagulants, anti-cardiolipin, and anti-β2 glycoprotein I antibodies).^[11]^ The following clinical variables were also registered: age, gender, body mass index (BMI), smoking habit, disease duration, rheumatoid factor (RF), anticitrullinated protein antibodies (ACPA), the presence of extra-articular manifestations, joint surgery, and administered therapies. Glucocorticoids (GCs), conventional synthetic-, targeted synthetic-, biological-disease modifying anti-rheumatic drugs (DMARDs) were registered. For those patients who were treated during the follow-up with sequential therapies, the treatment category was codified according to the drug to which the patient was exposed for a longer period. GCs therapy was also categorised, either high dosage or low dosage, as previously reported.^[8]^

### 2.4 Patients eligibility criteria

Patients were selected among those attending our Rheumatologic Clinic between January 2010 and December 2020. Patients fulfilling ACR/EULAR criteria for RA were considered in our evaluation if followed-up for at least 12 months. Each patient was investigated for VTE occurrence.

### 2.5 Patients exclusion criteria

Patients with a previous history of VTE were excluded to avoid an overestimation of VTE rate in our cohort.

### 2.6 Data sources, bias, and study size

Relevant data were collected by a review of clinical charts in the period between 2010 and 2020. Considering the retrospective design of our analysis, we tried to minimize possible biases with a careful definition of each variable to be assessed. A specific sample size was not estimated since this would be “real-life” evaluation of occurrence of VTE in patients with RA in our cohort.

### 2.7 Statistical methods

Statistics provided descriptive analysis of the data. Normally distributed continuous variables were expressed as mean ± standard deviation (SD), otherwise as median and range interquartile, as appropriate. The Shapiro-Wilk test was used to assess the normality of our continuous variable. Considering the relatively simple study design, we had a few missing data that we managed with their exclusion from the analysis. Incident cases were reported as incidence proportion and incidence rate per 1000 person-years at risk. Cox regression analyses were performed in exploiting the predictive role of some clinical variables on the likelihood of VTE occurrence. The Statistics Package for Social Sciences (SPSS for Windows, version 17.0, SPSS Inc., Chicago, IL) was used for all analyses.

### 2.8 Review of literature

We designed a search of literature on incidence of VTE in RA, by a review of reports published in indexed international journals until up March 2021. We followed proposed guidelines for preparing biomedical narrative review.^[12]^ MedLine (via PubMed) was searched for articles to be included. The bibliography of relevant articles was also hand-searched for identification of other potentially suitable studies.

## 3 Results of cohort study

In this evaluation, 347 consecutive patients, attending our Rheumatologic Clinic from 2010 to 2020, were studied. Patients with a previous history of VTE were excluded from this evaluation to avoid a possible overestimation of VTE rate. Assessed patients were mostly women (87.6%) with a mean age of 66.1 ± 11.3 years, as shown in Table 1. The median disease duration was 5 years (range interquartile 28) and 66.1% displayed the positivity for RF and/or ACPA. During the follow-up, 73.9% of patients were treated with low GCs, 69.7% with methotrexate, and 47.1% with biologic DMARDs (35.3% with tumor necrosis factor inhibitor [TNFi], 11.8% non-TNFi) and 4.2% with targeted synthetic DMARDs. Moreover, 28 out of 347 (11.5%) had extra-articular manifestations, 20 patients were affected by a secondary Sjogren syndrome. Interstitial lung disease (ILD) was identified in 5 patients whereas 3 had a leukocytoclastic vasculitis. The incidence proportion of VTE was 3.7% (2.7–4.7), occurring in 13 out of 347 patients. Considering over 1654 person-years, an incidence rate of 7.8 × 1000 (2.5–11.7) person-years was also estimated. The characteristics of patients with VTE were summarized in Table 1. We also performed an exploratory analysis comparing our results with previous data on general population^[3]^ in which VTE was estimated in occurring in 1 × 1000 person-years. In our cohort of RA patients, our incidence rate resulted to be significantly higher than general population (*P* < .001).

**Table 1:** Demographic and clinical features of the evaluated patients.

Patients	Three hundred forty seven patients	Thirteen patients with VTE
Gender (female/male)	87.6%/12.4%	84.6%/15.4%
ACR/EULAR 2010 classificative criteria	100.0%	100%
Age, mean ± SD, yr	66.1 ± 11.3	59.3 ± 10.4
Disease duration at the first observation, median (range), yr	5 (28)	6 (7)
RF and/or ACPA	66.1%	61.5%
Extra-articular disease	11.5%	7.6%
Smoking habit	47.8%	69.2%
GCs low dose	73.9%	76.9%
GCs high dose	26.1%	23.1%
MTX	69.7%	69.2%
LEF	17.6%	23.1%
SSZ	9.5%	0%
HCQ	18.1%	7.7%
Biologic DMARDs	47.1%	69.2%
TNFi	35.3%	35.9%
Non-TNFi	11.8%	33.3%
Targeted synthetic DMARDs	2.8%	0%

ACPA = anti-citrullinated protein antibody, DMARD = disease-modifying anti-rheumatic drug, GCs = glucocorticoids, HCQ = hydroxychloroquine, LEF = leflunomide, MTX = methotrexate, RF = rheumatoid factor, SSZ = sulfasalazine, TNFis = tumor necrosis factor.

In addition, we performed regression analyses to exploratively evaluate the predictive role of some selected variables on the risk of VTE occurrence. However, based on the relatively low number of VTE episodes in our cohort, only univariate hazard ratio (HR) analyses were exploited to assess possible predictive factors, as reported in Table 2. An increased age (HR 1.07, 95% confidence interval [CI] 1.01–1.14, *P* = .015), a higher BMI (HR 1.37, 95% CI 1.04–1.80, *P* = .026), and a longer disease duration (HR 1.11, 95% CI 1.03–1.20, *P* = .006) resulted to be significant predictors of VTE occurrence during the follow-up. On the contrary, sex, smoking habit, the presence of ACPA and/or RF, joint surgery, conventional synthetic DMARDs, biologic DMARDs, and targeted synthetic DMARDs did not predict VTE, as shown in Table 2. The most common administered treatment of acute VTE in all our patients was subcutaneous low molecular weight adjusted heparin; all patients discontinued anticoagulation after 3 months according to international guidelines.^[4]^ No patient developed any recurrence of VTE. Finally, thrombophilia screening tests did not report coagulation abnormalities and/or the presence of antiphospholipid antibodies. In addition, none of these patients were treated with oral contraceptives therapy. Considering the findings that no VTE recurrence was observed, the negativity of thrombophilia screening tests for coagulation abnormalities and/or for the presence of antiphospholipid antibodies, we supposed that VTE episodes were mainly related to RA.

**Table 2:** Cox regression univariate analysis assessing possible predictors of VTE in our cohort of RA patients.

Clinical variables	HR	95% CI	*P*-values
Age	1.07	1.01–1.14	.015
Gender	1.39	0.18–10.74	.750
Smoking habit	3.00	0.828–10.93	.094
BMI	1.37	1.04–1.81	.026
ACPA and/or RF	0.85	0.26–2.28	.790
Disease duration	1.13	1.03–1.20	.006
Joint surgery	0.05	0–34.17	.658
MTX	0.66	0.20–2.18	.500
Biologic DMARDs	0.85	0.24–2.95	.970
TNFi	0.93	0.30–2.89	.900
Non-TNFi	0.95	0.21–4.30	.947
Targeted synthetic DMARDs	0.48	0.00–89.69	.679

ACPA = anti-citrullinated protein antibody, BMI = body mass index, CI = confidence interval, DMARD = disease-modifying anti-rheumatic drug, HR = hazard ratio, MTX = methotrexate, RF = rheumatoid factor, TNFis = tumor necrosis factor.

## 4 Review of the available literature

As reported in Table 3, comparing our results with previous data derived from RCTs and administrative data, some different findings were retrieved about incidence of VTE.^[3,6,7,13–16]^ In this context, Matta et al^[15]^ evaluated incidence of VTE in patients discharged from short-stay hospitals in the United States between 1979 and 2005 who did not have surgery. Deep venous thrombosis was diagnosed in 1.64% of RA patients and 0.86% of controls, assessing almost 5 million RA patients and nearly 1 billion controls were studied.^[15]^ Liang et al^[16]^ reported similar results within the Rochester cohort of RA patients. The estimated incidence rate of VTE was 7.2% in 609 RA patients, an increased risk than 3.5% observed in controls.^[16]^ Furthermore, Ogdie et al^[13]^ examined the risk of incident VTE among patients with psoriasis, psoriatic arthritis, and RA. In this analysis, 12,084 psoriatic arthritis patients, 51,762 RA patients, and 194,288 psoriasis patients were identified and matched with 1,225,571 controls. RA patients showed the highest risk of VTE (No DMARD HR 1.29, 95% CI 1.18–1.39; DMARD HR 1.35, 95% CI 1.27–1.44) than other diseases.^[13]^ Subsequently, White^[3]^ reported a prospective cohort study to assess the occurrence of VTE in 39372 RA patients with follow-up from 1997 through 2010. RA patients had a higher risk of VTE than matched general population (incidence rate 5.9 [95% CI 5.1–6.6] vs 2.8 [95% CI 2.6–3.1] per 1000 person).^[3]^ Moreover, Molander et al^[6]^ performed a nationwide cohort study of the association between clinical RA disease activity and VTE risk. The authors reported 2241 incident VTE events within 1 year of follow-up, estimating a risk ratio for VTE in RA of 1.88 (95% CI 1.65–2.15) which paralleled with disease activity.^[6]^ Subsequently, a monitoring board of the ongoing Study A3921133 highlighted that the frequency of VTE and all-cause mortality was higher in patients receiving tofacitinib 10 mg twice daily than in patients treated with a TNFi. On these bases, Mease et al^[7]^ assessed 7964 RA patients treated with tofacitinib and an incidence rate of VTE was estimated to be 0.24 (0.15–0.37) and 0.26 (0.18–0.35). The vast majority of patients who experienced a VTE had multiple “traditional” risk factors, including older age, male sex, hypertension, and use of GCs, as frequently observed in RA patients.^[17–19]^ In addition, Desai et al^[14]^ evaluated the risk of VTE in RA patients on therapy with tofacitinib compared with TNFis identified from IBM “MarketScan” between 2012 and 2018, Medicare between 2012 and 2017, or Optum clinformatics between 2012 and 2019. A total of 42,201, 25,078, and 20,374 RA patients were identified in these databases. The incidence rate on 100 person-years were 0.42 (0.20–0.77) and 0.35 (0.29–0.42) in MarketScan, 1.18 (0.68–1.92) and 0.83 (0.71–0.97) in Medicare, and 0.19 (0.04–0.57) and 0.34 (0.26–0.44) in Optum for tofacitinib and TNFis, respectively.^[14]^ The occurrence of VTE in a total of 87,653 RA patients initiating tofacitinib or a TNFi was <1 per 100 person-years, without an increased risk of VTE comparing tofacitinib with TNFi.^[14]^

**Table 3:** Main results about VTE in RA from available literature.

Author	Cohort	Incidence proportion/incidence rate	Years	Main characteristics
Liang KP. et al	Retrospective medical record review	−7.2%	1955–1994	This is the first study to assess the incidence of noncardiac vascular disease in RA.
Matta F. et al	Non-Federal short-stay hospitals	−1.64%	1979–2005	Hospitalized patients with RA, the data suggested that RA could be a risk factor for VTE in hospitalized medical patients
Ogdie A. et al	Care medical record database in the UK	−4.2% in no DMARDs −4.7% in DMARDs −79.08 per 10,000 in no DMARDs −74.52 per per 10,000 in no DMARDs	1994–2014	Patients with PsA, RA, or psoriasis treated with or without DMARDs
Holmqvist ME et al	Prospective, population-based cohort study of 1 prevalent RA cohort	−3.5%/5.9 × 1000 person-years	1997–2010	RA cohort matched with general population
Molander V. et al	Swedish Rheumatology Quality Register (SRQ)	The overall cumulative 1-year incidence of VTE was 0.71% in the RA population	2006–2018	Patients with RA from the Swedish Rheumatology Quality Register. Strong association, with clinically relevant differences in absolute risks, between RA disease activity measured by DAS28 and the subsequent risk of VTE
Mease P. et al	This post-hoc analysis used data from separate tofacitinib RA, PsO, and PsA programmes.	−0.76%/ 0.25 per 100 person-year for RA −0.35%/ 0.14 per 100 person-year for PsO −0.25%/ 0.10 per 100 person-year for PsA	2012–2017 for RA 2015–2017 for PsO 2013–2017 for PsA	Tofacitinib-treated patients from the development programmes (RA: n = 7964; PsO: n = 3663; PsA: n = 783)
Desai RJ. et al	Cohort study using claims data from the IBM MarketScan, Medicare or Optum Clinformatics 2012–2019 databases.	Market Scan −0.42 per 100 person-years with tofacitinib −0.35 per 100 person-years with TNFi Medicare −1.18 per 100 person-years with tofacitinib −0.83 per 100 person-years with TNFi Optum −0.19 per 100 person-years with tofacitinib −0.34 per 100 person-years with TNFi	MarketScan 2012–2018 Medicare 2012–2017 Optum 2012–2019	This study evaluate the risk of (VTE) with tofacitinib compared to TNFI in RA patients

DMARD = disease-modifying anti-rheumatic drug, RA = Rheumatoid arthritis, TNFi = tumor necrosis factor inhibitor, VTE = venous thromboembolism.

## 5 Discussion

In our cohort, assessing the incidence of VTE in consecutive RA patients in a “real-life” setting, an incidence proportion of 3.7% (2.7–4.7) and an incidence rate of 7.8 × 1000 (2.5–11.7) person-years were estimated. Our findings paralleled previous experiences which reported a similar incidence of VTE. In fact, these studies had a similar design assessing a primary care medical record database and using hospitalization data.^[2,13]^ Conversely, our results may appear to be different from data derived from RCTs and administrative data, in which a higher prevalence was observed.^[7,14,16]^ These differences may be attributed with the specific methodology of these types of study. In fact, although RCTs are very relevant tools to assess the efficacy of a medical intervention, their applicability would be restricted to ideal conditions limiting their ability to portray what happens in “real-life” setting. Despite minimizing possible confounders, the selective inclusion criteria could limit the generalization of further extrapolated results in addition to the efficacy of studied medical interventions.^[16]^ Moreover, the administrative datasets may cover very large samples of patients for a period which are not achievable financially or logistically through any survey method.^[14]^ However, sometimes these data could be difficultly generalized because of lacking some documentation, due to confidentiality issues, and of adequate control variables.^[14]^ Conversely, the practice setting of a “real-life” study may increase the variability of the results, but it may more reliably in reproducing the complexity of the health care system than the controlled conditions in RCTs and administrative data.^[20]^ Taking together these observations, the need of further studies would be suggested, specifically designed, and adequately powered to investigate these issues.

In our cohort, we also assessed possible predictive factors of VTE occurrence. Our results paralleled with previous evidence about the predictive role of “traditional” cardiovascular (CV) risk factors.^[5]^ An increased incidence of VTE was associated with older age in our cohort. A higher prevalence of chronic diseases, a possible prolonged immobilization, and the “inflammageing” could contribute to VTE occurrence in the elderly.^[21,22]^ In addition, in older ages, obesity is more frequent, and this would be another important risk factor for VTE. Although the biological mechanisms are not fully understood, raised levels of fibrinogen and some clotting factors, low-grade systemic inflammation, raised intra-abdominal pressure and reduced venous return from the lower limbs are thought to be involved in enhancing VTE risk in obese patients.^[21,22]^ In our cohort, we also observed that a longer disease duration was a predictive factor for VTE occurrence. This result could be associated with an older age, but also with the burden of the disease over time with a longer exposition to an active inflammatory process. In addition, some specific RA features, such as the citrullination of fibrin within vasculature, could increase the risk of VTE in RA patients.^[23]^ Taking together these points, it is possible to speculate that VTE risk in RA is increased by a synergy between “traditional” CV risk factors and rheumatoid pro-inflammatory process, as observed in CVD.^[24,25]^ In fact, the inflammation may modulate the thrombotic state by upregulating procoagulants, downregulating anticoagulants and fibrinolysis.^[26]^ These pathways could be amplified by the presence of “traditional” CV risk factors furtherly enhancing the pro-thrombotic state.^[27]^ In this context, the role of anti-rheumatic drugs is not fully elucidated yet. The results of our study did not show any predictive role of therapies on VTE occurrence, despite a beneficial role of biologic DMARDs on endothelial dysfunction and “traditional” CV risk factors.^[28–30]^ Considering the “real-life” design of our cohort, the drugs were not systematically administered, and the choice of medications was left to the physicians. Consequently a “confounding by indication” bias could impair the predictive role of therapies on VTE, due to the possibility that more intensive therapeutic strategies could be administered to those patients affected by a more aggressive disease.

In addition, the analysis of this topic would be furtherly complicated since the deleterious effects of some drugs, such as GCs, may be balanced by the positive effects on inflammation thus confounding this possible relationship.^[31]^ However, it must be pointed out that although the synthetic and biologic DMARDs has led to substantial reductions of the chronic use of nonsteroidal anti-inflammatory drugs and cyclooxygenase-2 selective inhibitors in the treatment of RA, the use of these drugs is still common as self-administration by the patients due to chronic joint pain. Thus, it is possible to speculate that the administration of nonsteroidal anti-inflammatory drugs and cyclooxygenase-2 selective inhibitors could increase the risk of CVD as well as VTE in RA as observed in the general population.^[32–35]^

Finally, it remains to be defined the clinical usefulness of already available prediction scores of VTE risk and its possible recurrence on RA. In fact, a crucial point would be the identification of patients at higher risk of VTE to be treated with prophylaxis strategies. However, considering the clinical scenario of these patients, associated with elevated levels of both pro-inflammatory and prothrombotic markers and an increased prevalence of traditional cardiovascular risk factors, a possible specific prediction model would be needed to fully evaluate this issue. In addition, a comprehensive evaluation of VTE status in the rheumatologic setting could be complex, expensive, and time-consuming and thus the identification of biomarkers accurately reflecting the cardiometabolic risk profile and the occurrence of extra-articular manifestations are still awaited.^[36,37]^

As observed in any retrospective study, different limitations may impair the present evaluation, and our results should be prudently generalized, suggesting the need for further studies to fully clarify these topics. Despite providing an evaluation into thrombotic risk associated with RA, we did not quantify the relative risk of VTE than matched healthy controls due to the specific study design involving our cohort of patients with RA and exploratively assessed this issue with an historical cohort. Considering that no patient has developed any recurrence of VTE, thrombophilia screening tests did not report coagulation abnormalities and/or the presence of antiphospholipid antibodies, we supposed that VTE episodes were mainly related to RA. However, the retrospective design of our study did not allow us to fully evaluate this issue, suggesting the need of further studies. As a further limitation, considering the relatively low number of VTE episodes in our cohort, only univariate analyses were exploratively performed in assessing the possible predictive role of some selected variables on VTE occurrence. In addition, the study design did not allow to fully assess the role of therapeutic strategies on VTE.

In conclusion, in our “real-life” cohort, VTE burden has been suggested in patients with RA. Comparing our results with previous data derived from RCTs and administrative data, some different findings were retrieved about incidence of VTE. Assessing predictive factors, older age, higher BMI, and longer disease duration resulted to be significant predictors of VTE occurrence during the follow-up. Furthermore, the clinical usefulness of already available prediction scores of VTE risk and its possible recurrence is to be fully assessed. It could be also possible to hypothesize the development of a specific score for RA in assessing this issue considering the specific disease characteristics. Taking together these observations, a further evaluation of VTE occurrence in RA on specific designed studies is needed to provide more generalizable results to the daily clinical practice.

## Author contributions

All the authors made substantial contributions to the conception or design of the work, including acquisition, analysis, and interpretation of data for the present work. All the authors were involved in drafting and critically revising the work. All the authors approved the final version of the manuscript and agreed to be accountable for all aspects of the work.

**Conceptualization:** Alessandro Conforti, Paola Cipriani, Piero Ruscitti.

**Data curation:** Alessandro Conforti, Onorina Berardicurti, Viktoriya Pavlych, Ilenia Di Cola, Piero Ruscitti.

**Formal analysis:** Alessandro Conforti, Piero Ruscitti.

**Investigation:** Alessandro Conforti, Onorina Berardicurti, Viktoriya Pavlych, Ilenia Di Cola, Piero Ruscitti.

**Methodology:** Paola Cipriani, Piero Ruscitti.

**Project administration:** Paola Cipriani, Piero Ruscitti.

**Resources:** Alessandro Conforti, Paola Cipriani, Piero Ruscitti.

**Software:** Paola Cipriani, Piero Ruscitti.

**Supervision:** Paola Cipriani, Piero Ruscitti.

**Validation:** Alessandro Conforti, Onorina Berardicurti, Viktoriya Pavlych, Ilenia Di Cola, Paola Cipriani, Piero Ruscitti.

**Visualization:** Alessandro Conforti, Onorina Berardicurti, Viktoriya Pavlych, Ilenia Di Cola, Paola Cipriani, Piero Ruscitti.

**Writing – original draft:** Alessandro Conforti, Onorina Berardicurti, Viktoriya Pavlych, Ilenia Di Cola, Paola Cipriani, Piero Ruscitti.

**Writing – review & editing:** Alessandro Conforti, Onorina Berardicurti, Viktoriya Pavlych, Ilenia Di Cola, Paola Cipriani, Piero Ruscitti.
